# The Prevalence of Adrenal Insufficiency in Individuals with Traumatic Spinal Cord Injury: A Systematic Review and Meta-Analysis

**DOI:** 10.3390/jcm14072141

**Published:** 2025-03-21

**Authors:** Ali Hosseinzadeh, Rangchun Hou, Roy Rongyue Zeng, Martín Calderón-Juárez, Benson Wui Man Lau, Kenneth Nai Kuen Fong, Arnold Yu Lok Wong, Jack Jiaqi Zhang, Dalinda Isabel Sánchez Vidaña, Tiev Miller, Patrick Wai Hang Kwong

**Affiliations:** 1 Faculty of Medicine, University of British Columbia, Vancouver, BC V6T 1Z3, Canada; ahosse02@student.ubc.ca; 2 Department of Rehabilitation Sciences, The Hong Kong Polytechnic University, Hong Kong SAR, China; yen-rangchun.hou@polyu.edu.hk (R.H.); roy-rongyue.zeng@polyu.edu.hk (R.R.Z.); benson.lau@polyu.edu.hk (B.W.M.L.); kenneth.fong@polyu.edu.hk (K.N.K.F.); arnold.wong@polyu.edu.hk (A.Y.L.W.); wai-hang.kwong@polyu.edu.hk (P.W.H.K.); 3 Faculty of Sciences, Universidad Nacional Autónoma de México, Ciudad de México 04510, Mexico; martincj@ciencias.unam.mx; 4 Hospital General de Zona No. 67, Instituto Mexicano del Seguro Social, Apodaca 66600, Mexico

**Keywords:** adrenal cortex hormones, adrenal insufficiency, adrenocorticotropic hormone, glucocorticoids, spinal cord injury

## Abstract

**Background/Objectives**: Spinal cord injury (SCI) causes profound autonomic and endocrine dysfunctions, giving rise to adrenal insufficiency (AI), which is marked by a reduction in steroid hormone production. Left unaddressed, SCI-related AI (SCI-AI) can lead to life-threatening consequences such as severe hypotension and shock (i.e., adrenal crisis). However, symptoms are often non-specific, making AI challenging to distinguish from similar or overlapping cardiovascular conditions (e.g., orthostatic hypotension). Additionally, the etiology of SCI-AI remains unknown. This review aimed to synthesize the current literature reporting the prevalence, symptomology, and management of SCI-AI. **Methods:** A systematic search was performed to identify studies reporting AI following the cessation of glucocorticoid treatments in individuals with traumatic SCI. A random-effects meta-analysis was conducted to investigate the overall prevalence of SCI-AI. **Results:** Thirteen studies involving 545 individuals with traumatic SCI, most with cervical level injuries (n = 256), met the review criteria. A total of 4 studies were included in the meta-analysis. Primary analysis results indicated an SCI-AI pooled prevalence of 24.3% (event rate [ER] = 0.243, 95% confidence interval [CI] = 0.073–0.565, n = 4). Additional sensitivity analyses showed a pooled prevalence of 46.3% (ER = 0.463, 95%CI = 0.348–0.582, n = 2) and 10.8% (ER = 0.108, 95%CI = 0.025–0.368, n = 2) for case–control and retrospective cohort studies, respectively. High-dose glucocorticoid administration after SCI as well as the injury itself appear to contribute to the development of AI. **Conclusions:** The estimated prevalence of AI in people with traumatic SCI was high (24%). Prevalence was also greater among individuals with cervical SCI than those with lower-level lesions. Clinicians should be vigilant in recognizing the symptomatology and onset of SCI-AI. Further research elucidating its underlying pathophysiology is needed to optimize glucocorticoid administration for remediating AI in this vulnerable population.

## 1. Introduction

Spinal cord injury (SCI) is a debilitating neurological condition affecting 20.6 million people worldwide [1], with an incidence rate of roughly 40–45 per million people according to recent estimates [2]. The global incidence of SCI has also increased dramatically (52.7%) over a 30-year period (1990–2019), with traumatic injuries from falls and automobile accidents constituting the largest proportion of cases (approximately 707,000 in 2019) [1]. SCI causes a broad range of health-related consequences (e.g., sensorimotor impairment, cardiovascular dysfunction, neurogenic bowel and bladder) [3,4,5]. SCI can also alter immune and endocrinological function [6], with the severity of dysfunction being directly related to the level of injury [7]. Individuals with complete cervical and high thoracic SCI (i.e., above the 6th thoracic segment) are also considered to be at higher risk of developing life-threatening complications such as autonomic dysreflexia [8,9] and adrenal insufficiency (AI), a condition characterized by reduced production of adrenal corticosteroids (i.e., glucocorticoids, mineralocorticoids, and adrenal androgens) [10]. While autonomic dysreflexia is reported to occur in roughly half of individuals with high-level injuries [11], the prevalence of AI following traumatic SCI (SCI-AI) remains unknown.

AI has non-specific symptoms, such as fatigue, hypotension, and anorexia, as well as classical laboratory presentations such as hyperkalemia, hyponatremia, and hypoglycemia [12,13]. The secretion of corticosteroids is regulated by the hypothalamic–pituitary–adrenal (HPA) axis [14], and stressors such as cold, infection, and hemorrhage stimulate the hypothalamus to release more corticotropin-releasing hormone (CRH). CRH then triggers the pituitary gland to secrete adrenocorticotropic hormone (ACTH), which travels to the adrenal cortex, promoting the release of corticosteroids. This system is also modulated by negative feedback mechanisms at the pituitary and hypothalamic levels. Therefore, dysfunction at any level may lead to AI. Depending on the affected organ, AI can be defined according to primary (adrenal cortex), secondary (pituitary), or tertiary locations (hypothalamus) [15].

AI often presents in critically ill patients, and timely treatment has been shown to decrease the mortality rate by up to 10% [16]. However, AI is often overlooked in individuals with SCI [17]. This may be due to overlapping symptomatology between AI and common cardiovascular sequelae associated with traumatic SCI (e.g., orthostatic hypotension) [18]. People with SCI may present with profound resting hypotension, fatigue, and weakness, which are synonymous with typical AI symptoms [18,19]. Conversely, some individuals with SCI do not exhibit classical AI presentations (i.e., hyperkalemia, hyponatremia, hypoglycemia), thus confounding diagnostic accuracy [12]. Furthermore, the administration of high-dose glucocorticoids after acute SCI is also controversial. Although current guidelines do not support the use of high-dose glucocorticoids (e.g., methylprednisolone) after SCI, clinicians may opt to administer these at their discretion [20]. Following glucocorticoid dosage reduction or cessation, the adrenal glands may not produce glucocorticoid hormone promptly or in sufficient quantity, potentially causing AI [15]. Despite the inherent risk that AI poses, a comprehensive review of the existing literature that examines the prevalence, symptomatology, and management of this condition following SCI is currently lacking. In an effort to address the aforementioned knowledge gap and guide future research efforts, this review aimed to synthesize the current body of literature reporting the prevalence, symptoms, and management of AI following traumatic SCI. As the etiology and pathophysiology of SCI-related AI remains understudied, this review also aimed to elucidate potential mechanisms that lead to its development in this population.

## 2. Materials and Methods

### 2.1. Search Strategy

This review was prospectively registered in PROSPERO (CRD42024575188) and conducted in accordance with the Preferred Reporting Items for Systematic Review and Meta-Analysis (PRISMA) criteria, the Meta-analysis Of Observational Studies in Epidemiology (MOOSE) checklist, and Cochrane Handbook guidelines [21,22,23]. A systemic search for published studies reporting AI in people with traumatic SCI was performed independently by 2 authors using MEDLINE, Excerpta Medica dataBASE (EMBASE), Cumulative Index to Nursing and Allied Health Literature (CINAHL)-complete, Scopus, and Web of Science Core Collection databases from their respective inceptions to 18 November 2024. The concept domains “spinal cord injury” and “adrenal insufficiency” were used as thematic frameworks for developing the search strategy syntax. Citation management software (Zotero 7, Corporation for Digital Scholarship, Fairfax, VA, USA, https://www.zotero.org (accessed on 18 November 2024)) was used to archive and organize all database search results and assist in duplicate citation removal. Database search results were then exported to the Rayyan research collaboration platform (Rayyan Systems Inc., Cambridge, MA, USA, https://www.rayyan.ai (accessed on 18 November 2024)) for title and abstract screening. Inter-rater agreement for screened inclusions was determined using Cohen’s kappa (κ), with values of <0.00, 0.01–0.20, 0.21–0.40, 0.41–0.60, 0.61–0.80, and 0.81–1.00 representing poor (i.e., less than chance), slight, fair, moderate, substantial, and near perfect agreement between raters [24,25]. Discrepancies arising between reviewers were resolved through consultation with a third author. Reference lists of articles included as full-text were manually (i.e., by hand) searched for additional articles of relevance. Missing or omitted information and/or data relevant to the review were requested from the authors of included articles and added to the synthesis contingent on a reply within 14 days. The search strategy syntax used for each database is provided in File S1.

### 2.2. Inclusion/Exclusion Criteria

Articles published in English reporting AI following the cessation of glucocorticoid treatments in populations with acute, subacute, and chronic SCI were considered for inclusion (irrespective of study design). Studies involving non-traumatic SCI due to degenerative conditions (e.g., degenerative disk disease, spondylosis), vascular issues (e.g., spinal cord infarction, arteriovenous malformations), infections (e.g., spinal tuberculosis, epidural abscess), inflammatory/autoimmune disorders (e.g., multiple sclerosis, transverse myelitis, sarcoidosis), tumors (e.g., meningiomas, gliomas, metastatic cancers), genetic/congenital disorders (e.g., adrenoleukodystrophy, spina bifida), metabolic/endocrine disorders (e.g., vitamin B12 deficiency, Addison’s disease), or other conditions (e.g., Amyotrophic Lateral Sclerosis [ALS], Spinal Muscular Atrophy [SMA]) were excluded. Conference abstracts, theses, dissertations, and book chapters were also excluded.

### 2.3. Quality Appraisal

Study quality was assessed by 2 independent authors using The Joanna Briggs Institute (JBI) Critical Appraisal Checklist tools for case reports (8 items), case-series (10 items), case–control (10 items), and cohort studies (11 items) (https://jbi.global/critical-appraisal-tools (accessed on 18 November 2024)) [26]. For each item, a “yes” judgment was assigned if the criterion was satisfied. Low, moderate, and high quality ratings were given to studies according to the overall proportion of items meeting ≤50%, 50–70%, and ≥70% of the criteria, respectively.

### 2.4. Data Extraction

Data extraction was conducted by two authors independently, where one author extracted the data and the second author validated the extracted data to ensure accuracy. Inter-rater agreement was determined using Cohen’s κ. Data extracted were participant characteristics (age, sex, neurological level (NLI), and severity of injury using the American Spinal Injury Association Impairment Scale (AIS), and time since injury (TSI), type and dosage of glucocorticoid used, and characterization of AI (onset following cessation of glucocorticoids, location [i.e., primary, secondary, and tertiary AI], and resultant symptomatology).

### 2.5. Data Synthesis and Analyses

For the qualitative synthesis, extracted data were tabulated and reported narratively. For the quantitative synthesis, meta-analyses were conducted for groups of 2 or more studies using Comprehensive Meta-analysis software (CMA version 4.0, Biostat Inc., Englewood, NJ, USA). A random-effects model was chosen based on the assumption that AI prevalence would vary across populations, settings, and study designs [27]. Higgins’ I^2^-statistic was used to determine the proportion of variance in prevalence estimates between studies (i.e., variance in true occurrence relative to sampling error/chance) [28]. Conventionally, I^2^ has been used to indicate trivial, moderate, substantial, and considerable heterogeneity according to values of <25%, 25–50%, 50–75%, and >75%, respectively [21]. Reported event rates (ER) for SCI-AI were used to generate pooled prevalence estimates with 95% confidence intervals (95%CI). Percentage prevalence from each study was converted to raw prevalence scores, and separate primary meta-analyses were conducted to estimate AI prevalence using the DerSimonian–Laird method. Subsequent sensitivity analyses were performed by aggregating studies according to study design (i.e., case–control, retrospective cohort). For groups of ≥3 studies, absolute estimates of heterogeneity were expressed as 95% prediction intervals (95%PI) [28]. Meta-regression with Hartung–Knapp–Sidik–Jonkman adjustment was also performed to examine the influence of moderating factors (i.e., study design as a model covariate) on estimated prevalence [29]. Between-study variance and heterogeneity were determined using Tau(*τ*)^2^ (i.e., variance in true effect), Cochran’s Q-statistic, and degrees of freedom (df) (i.e., Q > df indicating a common effect). Additional subgroup analyses based on demographic and SCI-specific characteristics (e.g., age, sex, time since injury, neurological level, and severity of injury, etc.) and other potential moderating factors (i.e., AI onset, symptomatology) were underpowered. To the knowledge of the authors, there is no consensus regarding the estimated prevalence of adrenal conditions following traumatic SCI. According to a recent meta-analysis, the estimated global incidence of traumatic SCI is approximately 26.48 per million people (i.e., 0.002648%) [2]. By comparison, the proportion of individuals with adrenal dysfunction and disease among the general population resulting in primary, secondary, and tertiary AI is estimated to range between 6 per million people (i.e., 0.0006%) [30], with prevalence estimates ≤5%, 5–10%, and ≤10% indicating low, moderate and high prevalence, respectively [31]. Thus, the pooled prevalence was assumed to fall within the aforementioned range and was interpreted accordingly.

## 3. Results

### 3.1. Screening

**Figure 1:**
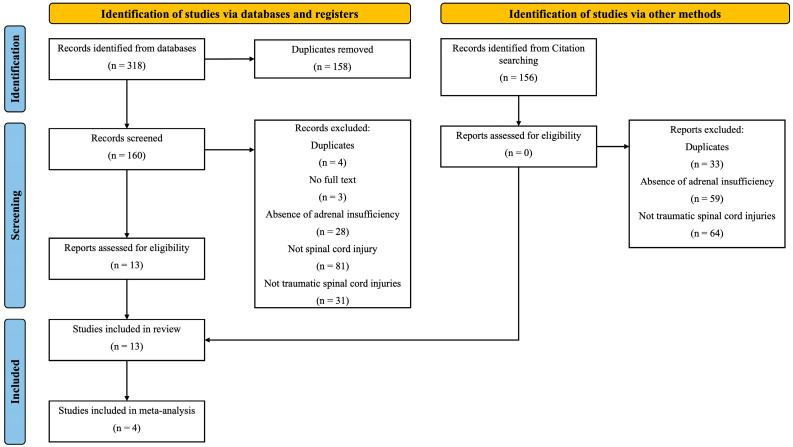
PRISMA flow diagram illustrating study identification, screening, and inclusion.

A total of 13 studies involving 545 individuals with traumatic SCI met the review criteria. Substantial inter-rater agreement was observed for screened inclusions (κ = 0.75). From these, 4 studies were included in the quantitative synthesis (Figure 1).

### 3.2. Study Quality

Study designs included case reports (n = 8), case series (n = 1), case–control (n = 2), and retrospective cohort studies (n = 2). Quality ratings were high for case reports (ranging from 6/8 [75%] to 8/8 [100%]), case series (7/10 [70%]), case–control studies (9/10 [90%]), and retrospective cohort studies (9/11 [81%] [16,17,32,33,34,35,36,37,38,39,40,41,42]. However, only 4 studies satisfied all quality criteria [17,35,37,40]. Four out of eight case reports did not describe adverse events, thereby reducing their quality [16,33,34,36]. One case report did not clearly describe the diagnostic tests used and treatment procedures followed [32]. One case series had relatively low quality on the basis of poorly defined inclusion criteria and the absence of participant demographic information [41]. One case–control study was rated unclear in terms of the use of a matched control group [42]. Two cohort studies similarly failed to identify possible confounding factors relating to the development of AI or measure exposure and/or responsiveness (i.e., following large-dose glucocorticoids and neurogenic shock) using tools with acceptable psychometric properties [38,39]. Quality ratings for each study are summarized in File S1 [43,44].

### 3.3. Participant Characteristics

Three studies did not report participant characteristics in detail [37,38,39]. Study participants included individuals with cervical (n = 256), thoracic (n = 7), or thoracolumbar (n = 41) SCI, and two healthy control groups (n = 42). Participants ranged from 15 to 81 years of age. Nine studies involved only male cohorts, while two studies involved only female cohorts. One study included both male and female participants. Across all included studies, only 4 out of 545 participants were female (0.007%). In terms of injury severity, four studies involved individuals with motor-complete SCI, four studies involved individuals with motor-incomplete SCI, and five studies did not specify injury completeness. SCI severity reported according to AIS ranged from A to D. A total of seven studies involved patients with acute SCI, and five studies involved individuals in the chronic phase of SCI recovery. Substantial inter-rater agreement was also observed for all extracted data (κ = 0.75). Participant characteristics are summarized in Table 1 [16,17,32,33,34,35,36,37,38,39,40,41,42].

### 3.4. Glucocorticoid Administration

In six studies, patients were taking glucocorticoids (dexamethasone, prednisone, and methylprednisolone) before the development of SCI-AI, as part of their standard treatment. However, treatment regimens varied. One study indicated individuals with SCI treated with high-dose glucocorticoids had a higher risk of developing AI than those who were not treated [38]. Two studies observed the accumulation of amyloid protein in the adrenal glands of patients with SCI during autopsy [32,37]. Hydrocortisone was the most common glucocorticoid used to treat SCI-AI among included studies [16,17,33,35,36,39].

### 3.5. Adrenal Insufficiency

The findings of this review suggest that AI in individuals with traumatic SCI may lead to symptoms such as low basal cortisol (13 studies), hypotension (8 studies), and fever (2 studies) (Table 1). Individuals with traumatic SCI lack the classic laboratory signs of AI (e.g., hyperkalemia and hyponatremia), making accurate diagnosis more difficult. The ACTH stimulation test was the most commonly used assessment of HPA-axis function. Out of the 13 included studies, 3 studies identified primary AI, 9 studies identified secondary or tertiary AI, and only 1 study did not specify AI type.

### 3.6. Analysis

**Figure 2:**
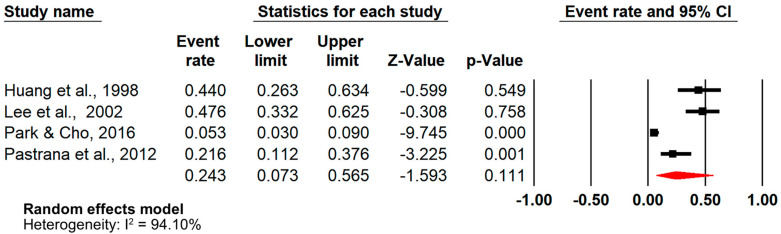
Forest plot (primary analysis) [37,38,39,42].

Results of the primary meta-analysis indicated an SCI-AI pooled prevalence of 24.3% (event rate [ER] = 0.243, 95%CI = [0.073–0.565], absolute heterogeneity: 95%PI = 0.004–0.996, n = 4) (Figure 2) [37,38,39,42].

**Figure 3:**
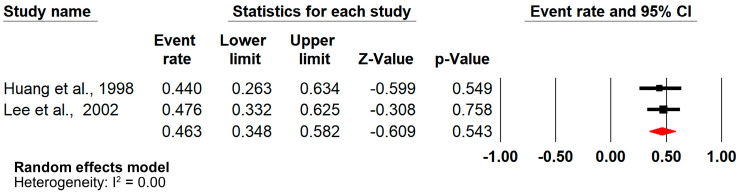
Forest plot (sensitivity analysis—case–control studies) [37,42].

**Figure 4:**
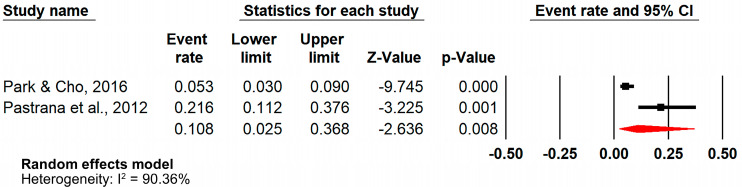
Forest plot (sensitivity analysis—retrospective cohort studies) [38,39].

Additional sensitivity analyses showed a pooled prevalence of 46.3% (ER = 0.463, 95%CI = [0.348–0.582], n = 2) (Figure 3) and 10.8% (ER = 0.108, 95%CI = [0.025–0.368], n = 2) (Figure 4) for case–control and retrospective cohort studies, respectively.

**Figure 5:**
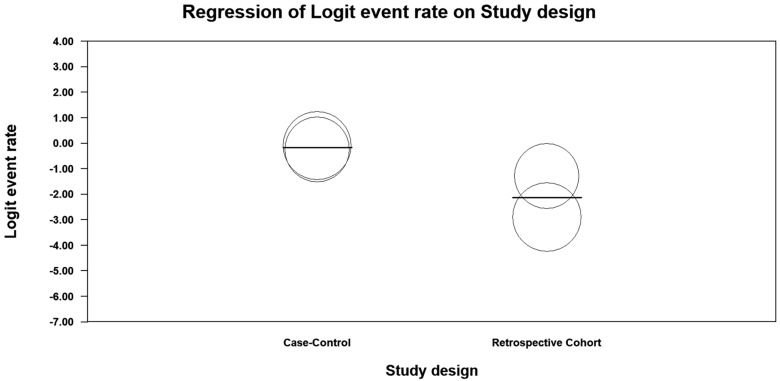
Scatterplot (meta regression).

Meta-regression results demonstrated that study design had a significant moderating effect on prevalence estimates (point estimate = 0.015, 95%CI = [−3.559–0.378], *p* < 0.001), explaining approximately 72% of the model variance (R^2^ analog = 0.72) (between-study variance and heterogeneity: *τ*^2^ = 1.909, Q = 50.83, df = 3, *p* < 0.001) (Figure 5).

## 4. Discussion

This review provides a comprehensive synthesis of the current literature reporting the prevalence, symptoms, and management of AI in individuals with traumatic SCI. The review findings indicate that the administration of high-dose glucocorticoids in the acute phase following SCI, as well as the SCI itself, may result in AI. Five studies found that AI in acute SCI patients occurred after the administration of glucocorticoids [16,17,33,35,38]. AI symptoms also appeared within two to four days after the cessation of high-dose glucocorticoids [17,35]. One study showed that 10 out of 12 SCI patients diagnosed with AI were treated with high-dose glucocorticoids. This supports the hypothesis that high-dose glucocorticoids may place patients with SCI at an increased risk of developing tertiary AI [38]. Notably, one individual with chronic SCI who experienced weight loss with the development of severe hypotension and tachycardia postoperatively after taking Megestrol acetate for 5 months was diagnosed with secondary AI [36]. Megestrol acetate, a synthetic progestin, likely exacerbated the occurrence of AI due to its glucocorticoid-like activity, which may inhibit the pituitary–adrenal axis [45]. While high-dose glucocorticoids were previously used to mitigate acute AI following SCI, recent research questions their neuroprotective effects in acute SCI and highlights adverse side effects such as hyperglycemia, lower respiratory tract infections, and gastrointestinal bleeding [46]. Therefore, caution is advised in the clinical use of high-dose glucocorticoids for patients with acute SCI.

The administration of glucocorticoids may potentially mask other physiological changes caused by SCI. Patients with acute SCI often experience severe stress and acute systemic inflammatory responses [47]. The complex pathophysiology of acute SCI makes it challenging to identify the underlying causes leading to the development of AI. Hemodynamic changes and the accumulation of inflammatory factors post-SCI may affect the HPA axis and cortisol secretion [48]. Additionally, Pastrana et al. (2012) found a 22% incidence of AI in patients with neurogenic shock, which may have been linked to impaired sympathetic activity resulting in vasodilation and hypotension. These conditions may also lead to inadequate adrenal perfusion and disrupted cortisol synthesis [39]. The reported prevalence of SCI-AI in mainly cervical and thoracic level injuries also suggests a possible association between AI and common cardiovascular conditions associated with SCI (e.g., autonomic dysreflexia, orthostatic hypotension), which often occur in individuals with lesions at or above the 6th thoracic segment. Further research is needed to explore potential mechanistic associations between these and other conditions post-SCI.

Clinicians should exercise caution when administering glucocorticoids to remediate AI in clinical practice. It is recommended that patients with SCI who have received glucocorticoid treatments at doses ≥ 120–150 mg/kg/day or who have undergone prolonged glucocorticoid therapy exceeding a duration of 10 consecutive days be monitored for AI following treatment cessation [17,33,35]. Additionally, the first week post-cessation may be an especially critical monitoring period, particularly among patients with complete cervical and high thoracic level SCI (at or above the 6th thoracic segment) who present with neurogenic shock [39]. The disruption of autonomic pathways, which causes loss of sympathetic tone and results in impaired catecholamine release subsequent to neurogenic shock, may also exacerbate electrolyte and fluid imbalance, alter normal hemodynamics (e.g., suboptimal venous return, hypotension), and cause profound bradycardia or dysrhythmia [39,49]. Therefore, routine monitoring of heart rate, blood pressure, serum potassium, and sodium, as well as symptoms potentially associated with AI (e.g., fever, fatigue, low basal cortisol), is strongly recommended. In addition to these critical surveillance parameters, definitive diagnostic evaluation through cortisol testing (e.g., ACTH stimulation test) is also advised when clinical suspicion of AI arises [50]. Although initial diagnosis and aftercare appear to be crucial in managing AI, a more protracted follow-up period involving routine monitoring of the aforementioned parameters is also advised given the elevated risk of rehospitalization and mortality within the first 2 years after diagnosis [51]. According to evidence from a recent retrospective study examining mortality risk and cause of death in individuals with AI, patients with secondary AI demonstrated a 52% increase in all-cause mortality risk within the first 1–2 years post-diagnosis compared to healthy individuals from a matched reference population [51]. Moreover, health-related consequences associated with delayed AI diagnosis can be life-threatening [39,51]. Patients with AI may experience a progressive deterioration in health, as well as an increased risk of developing adrenal crisis and other complications requiring invasive intervention (e.g., intubation, mechanical ventilation) [39]. The development of tailored guidelines for the early identification and clinical management of AI in people with traumatic SCI is warranted. Furthermore, underlying cardiovascular disease may substantially increase the risk of adrenal crisis-related death (Hazard Ratio = 1.54, 95%CI = 1.32–1.80) [51]. This is a key issue. According to previous epidemiological evidence, the risk of coronary artery disease (Adjusted Odds Ratio [OR] = 2.72, 95%CI = 1.94–3.82) and stroke (adjusted OR = 3.72, 95%CI = 2.22–6.23) increases by roughly 2- to 3-fold following SCI, respectively [52]. Research investigating the relationship between AI onset, persistence, severity, and underlying cardiovascular dysfunction in individuals with traumatic SCI is needed moving forward.

This review has several limitations. Most inclusions were either case reports or involved small sample sizes (n < 50), which may influence the pooled prevalence reported herein. Although the incidence of traumatic SCI is disproportionately higher among males than females (approximately 4:1) [2], most studies included in the meta-analyses involved exclusively male participant cohorts, thereby reducing the generalizability of the results. The population under study in this review was also limited to traumatic SCI. According to a recent meta-analysis, global incidence was substantially greater for traumatic than non-traumatic SCI cases (26.48 versus 17.93 cases per million people, respectively) [2]. The mechanism and pathophysiology of injury are also more diverse and complex among individuals with non-traumatic SCI. Moreover, AI onset and symptoms are also likely to differ substantially between cases with traumatic and non-traumatic SCI. Future studies are needed to examine AI prevalence, symptoms, and management among individuals with non-traumatic SCI due to degenerative conditions, vascular issues, infections, tumors, and autoimmune, genetic, congenital, metabolic, or endocrine disorders. Additionally, ACTH tests were the most common assessment used to identify AI. Although comparably safer than insulin intolerance tests and generally well-tolerated, the standardization (e.g., low-dose versus standard-dose, discrepancies in assay techniques), validation, and diagnostic accuracy of ACTH tests remain debated and inconclusive [50]. Inadequate reporting of participant demographics is another limitation of included studies. Several studies did not report medications used post-SCI (e.g., corticosteroids), which may have influenced the dose-dependent response between the glucocorticoids administered and the subsequent development of AI. Lastly, the meta-analyses were underpowered and should be interpreted with caution [53]. The results of the meta-regression also suggest a large variance in pooled prevalence estimates based on differences in study design. The large heterogeneity observed between studies is likely to be attributed to the methodological diversity of the studies included in the meta-analyses (i.e., case–control versus retrospective cohort) and population-based discrepancies between the two retrospective studies (i.e., one included all individuals with SCI, and the other only included SCI patients with neurogenic shock). Further research involving larger cohorts with similar clinical presentations is needed to gain a better understanding of SCI-AI epidemiology and its underlying pathophysiology.

## 5. Conclusions

The findings of this review suggest that the use of high-dose glucocorticoids in the acute phase following traumatic SCI may result in the development of AI. The general prevalence of AI among individuals with higher levels of injury (i.e., cervical and thoracic) also suggests a possible association between AI and cardiovascular dysfunction in this population. Clinicians should be vigilant in recognizing the onset of AI following SCI according to the symptomatology identified in this review (i.e., low basal cortisol, hypotension, and fever). Further research elucidating the underlying pathophysiology of AI is needed in order to optimize glucocorticoid administration for remediating AI in people with traumatic SCI.

## Figures and Tables

**Table 1:** Study/Participant Characteristics.

Author (Year)	Study Design	SCI-AI Prevalence	Event Rate	Participant Characteristics							Glucocorticoids/Mineralocorticoid			Adrenal Insufficiency		
				SCI (n = 545)/Control (n = 42)	Sex	Age	Neurological Level of Injury	Classification Based on AIS	Recovery Stage	Time Since Injury (Years)	Glucocorticoids or Mineralocorticoid (Before AI)	Name	Dose	Onset	Signs/Symptoms	SCI-AI Location/Level Affected
[32] (Baird-Howell & Wurzel, 2011)	Case report	N/A	N/A	SCI (n = 1)	Male	41	T2	N/A	Chronic	20	N/A	N/A	N/A	20 years after SCI (Admission: urinary tract infection)	Gastrointestinal bleeding, death, renal failure, amyloidosis	Primary
[33] (Garcia-Zozaya, 2006)	Case report	N/A	N/A	SCI (n = 1)	Male	21	C6	Grade A	Acute	N/A	Glucocorticoids	Methylprednisolone	Bolus 30 mg/kg over 15 min, with maintenance infusion of 5.4 mg/kg per hour for 23 h	2 weeks after SCI	Hypotension resistant to vasopressor and volume resuscitation therapy	Tertiary
[42] (Huang et al., 1998)	Case–control study	11/25	0.440	SCI (n = 25); Control (n =25)	Male	Range: 18–55 (mean: 35.4)	C5–C8 (n = 9) T1–L2 (n = 16)	N/A	Chronic	1.1–15.8 (mean 35.4)	N/A	N/A	N/A	N/A	N/A	Primary
[34] (Ishiki et al., 2024)	Case report	N/A	N/A	SCI (n = 1)	Female	34	C7	Grade C	Acute	N/A	N/A	N/A	N/A	12 days after SCI	No AI-related symptoms; hyperkalemia, slight hyponatremia	Secondary Pituitary
[35] (Lecamwasam et al., 2004)	Case report	N/A	N/A	SCI (n = 1)	Male	23	C5	N/A	Acute	N/A	Glucocorticoids	Dexamethasone, Methylprednisolone	Dexamethasone: 560 mg followed by 100 mg per hour for 6 h intravenously Methylprednisolone: 5.4 mg/kg per hour for 23 h intravenously	4 days after steroid cessation	fever, hypotension, low basal cortisol	Tertiary
[36] (Lee & Glenn, 2000)	Case report	N/A	N/A	SCI (n = 1)	Male	51	C5	Grade C	Chronic	8	A synthetic progestin with glucocorticoid-like activity	Megestrol acetate	200 mg by mouth, twice per day for 5 months	8 years after SCI (Admission: difficulty with bladder catheterization and left flank pain possibly caused by a left kidney stone)	Mild hypotension, sinus tachycardia, hypoglycemia, hyponatremia	Secondary
[37] (Lee et al., 2002)	Case–control study	20/42	0.476	SCI (n = 42); Control (n =17)	Male	Mean (SD): 40.5 (7.8)	N/A	Grade A or B	Chronic	>1	N/A	N/A	N/A	N/A	Relatively larger adrenal volume than healthy individuals	Secondary
[38] (Park & Cho, 2016)	Retrospective cohort	12/228 (Treated with large-dose glucocorticoid = 10; not treated with large-dose glucocorticoid = 2)	0.053	SCI (n = 228)	Patients diagnosed with AI: Male (n = 10), Female (n = 2)	Range: 20–81	Patients who have suspected AI: C3–C5 (n = 23) T6–T12 (n = 6) Patients diagnosed with AI: C3–C7 (n = 11) T10 (n = 1)	Patients diagnosed with AI: Grade A (n = 2), Grade C (n = 2), Grade D (n = 8)	NA	N/A	Glucocorticoids	N/A	Large dose	N/A	Fatigue, hypotension, anorexia	Secondary/Tertiary
[39] (Pastrana et al., 2012)	Retrospective cohort	8/37	0.216	SCI (n = 199) SCI patients with neurogenic shock (n = 37)	NA	Range: 18–66 (mean: 32.3)	All patients: Cervical level (n = 199) Patients diagnosed with AI: C4–C5 (n = 8)	Grade A (n = 8)	Acute	N/A	N/A	N/A	N/A	N/A	Low cortisol, hypotension, neurogenic shock	NA
[40] (Steinberg et al., 1978)	Case report	N/A	N/A	SCI (n = 1)	Male	15	C5	N/A	Acute	N/A	N/A	N/A	N/A	2 months after SCI	Hypercalcemia, orthostatic hypotension, low plasma cortisol level	Primary
[41] (Wang & Huang, 1999)	Case series (same data as Lee (2002), excluded from meta-analysis)	20/42	0.476	SCI (n = 42)	Male	Range: 20–60 (mean: 39.2)	All patient: Cervical level (n = 17) Thoracolumbar level (n = 25) Patients diagnosed with AI: C4–C6 (n = 7) T3–T12 (n = 13)	Grade A or B	Chronic	1.1–35 (mean 9.4)	N/A	N/A	N/A	N/A	Decreased adrenal reserve	Secondary
[16] (Weant et al., 2008)	Case report	N/A	N/A	SCI (n = 2)	Male	39, 75	Case 1: C6 Case 2: C1	N/A	Acute	N/A	Glucocorticoids	Case 1: Methylprednisolone; Case 2: N/A	N/A	Case 1: 23–31 days post-admission; Case 2: Day 6	Case 1: low cortisol, fever;Case 2: hypotensive, not responsive to vasopressors	Secondary/Tertiary
[17] (Yang et al., 2014)	Case report	N/A	N/A	SCI (n = 1)	Female	61	C3	Grade D	Acute	N/A	Glucocorticoids	Dexamethasone	Dexamethasone intravenously for 11 days: 4 mg every 6 h for 18 doses 2 mg every 6 h for 5 doses 2 mg every 12 h for 7 doses	2 days after steroid cessation	Low basal cortisol, acute neck pain, fatigue, muscle weakness, hypotension	Tertiary

Abbreviation: AIS: American Spinal Injury Association Impairment; SCI-AI: spinal cord injury-related adrenal insufficiency.

## Data Availability

All data are presented within the manuscript and accompanying tables and figures.

## Supplementary Materials

The following supporting information can be downloaded at https://www.mdpi.com/article/10.3390/jcm14072141/s1: File S1: Section S1: Search Strategy; Section S2: Quality appraisal [54,55,56,57].

## Abbreviations

The following abbreviations are used in this manuscript:

ACTH	Adrenocorticotropic hormone
AI	Adrenal insufficiency
AIS	American Spinal Injury Association Impairment Scale
ALS	Amyotrophic lateral sclerosis
CRH	Corticotropin-releasing hormone
ER	Even rate
HPA	Hypothalamic–pituitary–adrenal
NLI	Neurological level of injury
OR	Odds ratio
SCI	Spinal cord injury
SCI-AI	Spinal cord injury-related adrenal insufficiency
SMA	Spinal muscular atrophy
TSI	Time since injury
95%CI	95% confidence intervals
95%PI	95% prediction intervals
