# Supplementary material for: The Prevalence of Adrenal Insufficiency in Individuals with Traumatic Spinal Cord Injury: A Systematic Review and Meta-Analysis
Source: J Clin Med. 2025 Mar 21;14(7):2141. doi: 10.3390/jcm14072141 (PMC11989398; doi:10.3390/jcm14072141)
Supplement: Supplementary file 1 [file jcm-14-02141-s001.zip › jcm-3514450-supplementary.pdf]

Section S1

Search Strategy

Date of database search: Monday 11NOV24
TOTAL NUMBER OF ARTICLES (all databases): **318**

MEDLINE (via EbscoHost)

ID	Database settings	Search term<br>Spinal cord injury	Search Strategy	No. of articles
1	MM	Spinal cord injury	MM "Spinal Cord Injuries"	40620
2	TI OR AB		TI ((spinal cord) N2 (injur*)) OR AB ((spinal cord) N2 (injur*))	52576
3			TI "SCI" OR AB "SCI"	45551
4	TI OR AB	Quadriplegi*	TI quadriplegi* OR AB quadriplegi*	4633
5	TI OR AB	Tetraplegi*	TI tetraplegi* OR AB tetraplegi*	4907
6	TI OR AB	Paraplegi*	TI paraplegi* OR AB paraplegi*	19085
7	1 OR 2 OR 3 OR 4 OR 5 OR 6			101989
		Adrenal insufficiency		
8	MM	Adrenal insufficiency	MM "adrenal insufficiency"	5265
9	TI OR AB	Adrenal Gland insufficiency<br>Adrenal cortex insufficiency<br>Adrenocortical insufficiency	TI ((adren*) N1 (insufficienc*)) OR AB ((adren*) N1 (insufficienc*))	8891
10	TI OR AB	Adrenal hypofunction<br>Adrenal cortex hypofunction	TI ((adren*) N1 (hypofunction)) OR AB ((adren*) N1 (hypofunction))	193
11	TI OR AB	Adrenal failure	TI ((adren*) N1 (failure)) OR AB ((adren*) N1 (failure))	1015
12	TI OR AB	Adrenal crisis	TI ((adren*) N1 (crisis)) OR AB ((adren*) N1 (crisis))	1115
13	TI OR AB	Addison's disease<br>Addisons disease	TI ((addison*) N1 (disease)) OR AB ((addison*) N1 (disease))	3992

		Addison biermer disease		
14	TI OR AB	Addison crisis<br>Addisonian crisis	TI ((addison*) AND (crisis)) OR AB ((addison*) AND (crisis))	465
15	TI OR AB	Hypoadrenalism<br>Hypoadrenia<br>Hypoadrenocorticism	TI hypoadren* OR AB hypoadren*	966
16	TI OR AB	Hypocortisolism	TI hypocortisolism OR AB hypocortisolism	590
17	8 OR 9 OR 10 OR 11 OR 12 OR 13 OR 14 OR 15 OR 16			16237
18	Total number	7 AND 17	Filter: English	46

Scopus

ID	Database settings	Search term<br>Spinal cord injury	Search Strategy	No. of articles
1	TI OR AB OR KEY	Spinal cord injury	TITLE-ABS-KEY(spinal cord injury)	110171
2			TITLE-ABS-KEY((spinal cord) W/2 (injur*))	87972
3			TITLE-ABS-KEY(SCI)	107741
4	TI OR AB	Quadriplegi*	TITLE-ABS(quadriplegi*)	6126
5	TI OR AB	Tetraplegi*	TITLE-ABS(tetraplegi*)	6071
6	TI OR AB	Paraplegi*	TITLE-ABS(paraplegi*)	24112
7	1 OR 2 OR 3 OR 4 OR 5 OR 6			213359
		Adrenal insufficiency		
8	TI OR AB OR KEY	Adrenal insufficiency	TITLE-ABS-KEY(adrenal insufficiency)	22952
9		Adrenal Gland insufficiency<br>Adrenal cortex insufficiency<br>Adrenocortical insufficiency	TITLE-ABS-KEY((adren*) W/1 (insufficienc*))	20930
10		Adrenal hypofunction<br>Adrenal cortex hypofunction	TITLE-ABS-KEY((adren*) W/1 (hypofunction))	2109

11		Adrenal failure	TITLE-ABS-KEY((adren*) W/1 (failure))	1274
12		Adrenal crisis	TITLE-ABS-KEY((adren*) W/1 (crisis))	1309
13		Addison's disease<br>Addisons disease<br>Addison biermer disease	TITLE-ABS-KEY((addison*) W/1 (disease))	8787
14		Addison crisis<br>Addisonian crisis	TITLE-ABS-KEY((addison*) W/1 (crisis))	416
15	TI OR AB	Hypoadrenalism<br>Hypoadrenia<br>Hypoadrenocorticism	TITLE-ABS(hypoadren*)	1167
16	TI OR AB	Hypocortisolism	TITLE-ABS(hypocortisolism)	624
17	8 OR 9 OR 10 OR 11 OR 12 OR 13 OR 14 OR 15 OR 16			31379
18	Total number	7 AND 17	Filter: English	113

Web of Science Core Collection

ID	Database settings	Search term<br>Spinal cord injury	Search Strategy	No. of articles
1	TOPIC	Spinal cord injury	TS=(spinal cord injury)	86271
2			TS=((spinal cord) NEAR/2 (injur*))	69155
3			TS=(SCI)	108484
4	TI OR AB	Quadriplegi*	TI=(quadriplegi*) OR AB=(quadriplegi*)	4125
5	TI OR AB	Tetraplegi*	TI=(tetraplegi*) OR AB=(tetraplegi*)	4423
6	TI OR AB	Paraplegi*	TI=(paraplegi*) OR AB=(paraplegi*)	16021
7	1 OR 2 OR 3 OR 4 OR 5 OR 6			189091
		Adrenal insufficiency		
8	TOPIC	Adrenal insufficiency	TS=(adrenal insufficiency)	9738
9	TI OR AB	Adrenal Gland insufficiency<br>Adrenal cortex insufficiency<br>Adrenocortical insufficiency	TI=((adren*) NEAR/1 (insufficienc*)) OR AB=((adren*) NEAR/1 (insufficienc*))	7395

10	TI OR AB	Adrenal hypofunction<br>Adrenal cortex hypofunction	TI=((adren*) NEAR/1 (hypofunction)) OR AB=((adren*) NEAR/1 (hypofunction))	113
11	TI OR AB	Adrenal failure	TI=((adren*) NEAR/1 (failure)) OR AB=((adren*) NEAR/1 (failure))	802
12	TI OR AB	Adrenal crisis	TI=((adren*) NEAR/1 (crisis)) OR AB=((adren*) NEAR/1 (crisis))	931
13	TI OR AB	Addison's disease<br>Addisons disease<br>Addison biermer disease	TI=((addison*) NEAR/1 (disease)) OR AB=((addison*) NEAR/1 (disease))	2412
14	TI OR AB	Addison crisis<br>Addisonian crisis	TI=((addison*) NEAR/1 (crisis)) OR AB=((addison*) NEAR/1 (crisis))	232
15	TI OR AB	Hypoadrenalism<br>Hypoadrenia<br>Hypoadrenocorticism	TI=(hypoadren*) OR AB=(hypoadren*)	880
16	TI OR AB	Hypocortisolism	TI=(hypocortisolism) OR AB=(hypocortisolism)	530
17	8 OR 9 OR 10 OR 11 OR 12 OR 13 OR 14 OR 15 OR 16			13789
18	Total number	7 AND 17	Filter: English	46

EMBASE

ID	Database settings	Search term<br>Spinal cord injury	Search Strategy	No. of articles
1		Spinal cord injury	'spinal cord injury'/exp OR 'spinal cord injury'	111302
2	TI OR AB		('spinal cord' NEAR/2 injur*):ab,ti	65861
3			SCI:ti,ab	65091
4	TI OR AB	Quadriplegi*	quadriplegi*:ab,ti	6484
5	TI OR AB	Tetraplegi*	tetraplegi*:ab,ti	6758
6	TI OR AB	Paraplegi*	paraplegi*:ab,ti	25354
7	1 OR 2 OR 3 OR 4 OR 5 OR 6			172362

		Adrenal insufficiency		
8	MM	Adrenal insufficiency	'adrenal insufficiency'/exp OR 'adrenal insufficiency'	22185
9	TI OR AB	Adrenal Gland insufficiency<br>Adrenal cortex insufficiency<br>Adrenocortical insufficiency	(adren* NEAR/1 insufficienc*):ab,ti	13624
10	TI OR AB	Adrenal hypofunction<br>Adrenal cortex hypofunction	(adren* NEAR/1 hypofunction):ab,ti	160
11	TI OR AB	Adrenal failure	(adren* NEAR/1 failure):ab,ti	1186
12	TI OR AB	Adrenal crisis	(adren* NEAR/1 crisis):ab,ti	1649
13	TI OR AB	Addison's disease<br>Addisons disease<br>Addison biermer disease	(addison* NEAR/1 disease):ab,ti	541
14	TI OR AB	Addison crisis<br>Addisonian crisis	(addison* NEAR/1 crisis):ab,ti	376
15	TI OR AB	Hypoadrenalism<br>Hypoadrenia<br>Hypoadrenocorticism	hypoadren*:ab,ti	1308
16	TI OR AB	Hypocortisolism	hypocortisolism:ab,ti	927
17	8 OR 9 OR 10 OR 11 OR 12 OR 13 OR 14 OR 15 OR 16			25976
18	Total number	7 AND 17	Filter: English	102

CINAHL Complete (via EbscoHost)

ID	Database settings	Search term<br>Spinal cord injury	Search Strategy	No. of articles
1	MM	Spinal cord injury	MM "Spinal Cord Injuries"	18275
2	TI OR AB		TI ((spinal cord) N2 (injur*)) OR AB ((spinal cord) N2 (injur*))	19523
3			TI "SCI" OR AB "SCI"	12490

4	TI OR AB	Quadriplegi*	TI “quadriplegi*” OR AB “quadriplegi*”	1394
5	TI OR AB	Tetraplegi*	TI “tetraplegi*” OR AB “tetraplegi*”	2074
6	TI OR AB	Paraplegi*	TI “paraplegi*” OR AB “paraplegi*”	4001
7	1 OR 2 OR 3 OR 4 OR 5 OR 6			30578
		Adrenal insufficiency		
8	MM	Adrenal insufficiency	MM “adrenal insufficiency”	901
9	TI OR AB	Adrenal Gland insufficiency<br>Adrenal cortex insufficiency<br>Adrenocortical insufficiency	TI ((adren*) N1 (insufficienc*)) OR AB ((adren*) N1 (insufficienc*))	1473
10	TI OR AB	Adrenal hypofunction<br>Adrenal cortex hypofunction	TI ((adren*) N1 (hypofunction)) OR AB ((adren*) N1 (hypofunction))	33
11	TI OR AB	Adrenal failure	TI ((adren*) N1 (failure)) OR AB ((adren*) N1 (failure))	119
12	TI OR AB	Adrenal crisis	TI ((adren*) N1 (crisis)) OR AB ((adren*) N1 (crisis))	249
13	TI OR AB	Addison’s disease<br>Addisons disease<br>Addison biermer disease	TI ((addison*) N1 (disease)) OR AB ((addison*) N1 (disease))	346
14	TI OR AB	Addison crisis<br>Addisonian crisis	TI ((addison*) AND (crisis)) OR AB ((addison*) AND (crisis))	72
15	TI OR AB	Hypoadrenalism<br>Hypoadrenia<br>Hypoadrenocorticism	TI hypoadren* OR AB hypoadren*	74
16	TI OR AB	Hypocortisolism	TI hypocortisolism OR AB hypocortisolism	84
17	8 OR 9 OR 10 OR 11 OR 12 OR 13 OR 14 OR 15 OR 16			2363
18	Total number	7 AND 17	Filter: English	11

Section S2

Quality appraisal

Yes (green) · No (red) · Unclear (yellow)

Author (year)	Q1	Q2	Q3	Q4	Q5	Q6	Q7	Q8	Q9	Q10	Q11	% Yes	Quality
**Case reports**													
Baird-Howell & Wurzel (2011)	Yes	Yes	Yes	Unclear	Unclear	Yes	Yes	Yes				75.0%	High
Garcia-Zozaya (2006)	Yes	Yes	Yes	Yes	Yes	Yes	No	Yes				87.5%	High
Ishiki et al. (2024)	Yes	Yes	Yes	Yes	Yes	Yes	No	Yes				87.5%	High
Lecamwasam et al. (2004)	Yes	Yes	Yes	Yes	Yes	Yes	Yes	Yes				100.0%	High
Lee & Glenn (2000)	Yes	Yes	Yes	Yes	Yes	Yes	No	Yes				87.5%	High
Steinberg et al. (1978)	Yes	Yes	Yes	Yes	Yes	Yes	Yes	Yes				100.0%	High
Weant et al. (2008)	Yes	Yes	Yes	Yes	Yes	Yes	No	Yes				87.5%	High
Yang et al. (2014)	Yes	Yes	Yes	Yes	Yes	Yes	Yes	Yes				100.0%	High
**Case series**													
Wang & Huang (1999)	Yes	Yes	Yes	Unclear	Unclear	No	Yes	Yes	Yes	Yes		70.0%	High
**Case control studies**													
Huang et al. (1998)	Yes	Unclear	Yes	Yes	Yes	Yes	Yes	Yes	Yes	Yes		90.0%	High
Lee et al. (2002)	Yes	Yes	Yes	Yes	Yes	Yes	Yes	Yes	Yes	Yes		100.0%	High
**Cohort studies**													
Park & Cho (2016)	Yes	Yes	Yes	No	No	Yes	Yes	Yes	Yes	Yes	Yes	81.8%	High
Pastrana et al. (2012)	Yes	Yes	Yes	No	No	Yes	Yes	Yes	Yes	Yes	Yes	81.8%	High

Figure S1. Quality assessment of included studies conducted using the appropriate JBI Critical Appraisal Checklist for each corresponding study design.

Specific items of ***<u>Case reports</u>*** Critical Appraisal JBI tools:

Q1: Were patient’s demographic characteristics clearly described?

Q2: Was the patient’s history clearly described and presented as a timeline?

Q3: Was the current clinical condition of the patient on presentation clearly described?

Q4: Were diagnostic tests or methods and the results clearly described?

Q5: Was the intervention(s) or treatment procedure(s) clearly described?

Q6: Was the post-intervention clinical condition clearly described?

Q7: Were adverse events (harms) or unanticipated events identified and described?

Q8: Does the case report provide takeaway lessons?

Specific items of ***<u>Case series</u>*** Critical Appraisal JBI tools:

Q1: Were there clear criteria for inclusion in the case series?

Q2: Was the condition measured in a standard, reliable way for all participants included in the case series?

Q3: Were valid methods used for identification of the condition for all participants included in the case series?

Q4: Did the case series have consecutive inclusion of participants?

Q5: Did the case series have complete inclusion of participants?

Q6: Was there clear reporting of the demographics of the participants in the study?

Q7: Was there clear reporting of clinical information of the participants?

Q8: Were the outcomes or follow-up results of cases clearly reported?

Q9: Was there clear reporting of the presenting site(s)/clinic(s) demographic information?

Q10: Was statistical analysis appropriate?

Specific items of ***Case control studies*** Critical Appraisal JBI tools:

Q1: Were the groups comparable other than presence of disease in cases or absence of disease in controls?

Q2: Were cases and controls matched appropriately?

Q3: Were the same criteria used for identification of cases and controls?

Q4: Was exposure measured in a standard, valid and reliable way?

Q5: Was exposure measured in the same way for cases and controls?

Q6: Were confounding factors identified?

Q7: Were strategies to deal with confounding factors stated?

Q8: Were outcomes assessed in a standard, valid and reliable way for cases and controls?

Q9: Was the exposure period of interest long enough to be meaningful?

Q10: Was appropriate statistical analysis used?

Specific items of ***Cohort studies*** Critical Appraisal JBI tools:

Q1: Were the two groups similar and recruited from the same population?

Q2: Were the exposures measured similarly to assign people to both exposed and unexposed groups?

Q3: Was the exposure measured in a valid and reliable way?

Q4: Were confounding factors identified?

Q5: Were strategies to deal with confounding factors stated?

Q6: Were the groups/participants free of the outcome at the start of the study (or at the moment of exposure)?

Q7: Were the outcomes measured in a valid and reliable way?

Q8: Was the follow up time reported and sufficient to be long enough for outcomes to occur?

Q9: Was follow up complete, and if not, were the reasons to loss to follow up described and explored?

Q10: Were strategies to address incomplete follow up utilized?

Q11: Was appropriate statistical analysis used?

References

Gagnier JJ, Kienle G, Altman DG, Moher D, Sox H, Riley D, CARE Group. The CARE Guidelines: Consensus Based Clinical Case Reporting Guideline Development. Headache: The Journal of Head and Face Pain, 2013;53(10):1541-1547.

Dekkers OM, Egger M, Altman DG, Vandenbroucke JP. Distinguishing case series from cohort studies. Annals of Internal Medicine. 2012;156(1 Part 1):37-40.

Esene IN, Ngu J, El Zoghby M, Solaroglu I, Sikod AM, Kotb A et al. Case series and descriptive cohort studies in neurosurgery: the confusion and solution. Child's Nervous System. 2014;30(8):1321-32.

Abu-Zidan FM, Abbas AK, Hefny AF. Clinical “case series”: a concept analysis. African Health Sciences. 2012;12(4):557-62.

Straus SE, Richardson WS, Glasziou P, Haynes RB. Evidence-based medicine: How to practice and teach EBM. 3rd Edition ed: Elsevier 2005.

Moola S, Munn Z, Tufanaru C, Aromataris E, Sears K, Sfetcu R, Currie M, Qureshi R, Mattis P, Lisy K, Mu P-F. Chapter 7: Systematic reviews of etiology and risk . In: Aromataris E, Munn Z (Editors). JBI Manual for Evidence Synthesis. JBI, 2020.
